# Resistant starch slows the progression of CKD in the 5/6 nephrectomy mouse model

**DOI:** 10.14814/phy2.14610

**Published:** 2020-10-10

**Authors:** Oleg Karaduta, Galina Glazko, Zeljko Dvanajscak, John Arthur, Samuel Mackintosh, Lisa Orr, Yasir Rahmatallah, Laxmi Yeruva, Alan Tackett, Boris Zybailov

**Affiliations:** ^1^ Department of Biochemistry and Molecular Biology UAMS Little Rock AR USA; ^2^ Department of Biomedical Informatics UAMS Little Rock AR USA; ^3^ Arkana Laboratories Little Rock AR USA; ^4^ Division of Nephrology UAMS Little Rock AR USA; ^5^ Proteomics Core Facility UAMS Little Rock AR USA; ^6^ Arkansas Children’s Nutrition Center Little Rock AR USA; ^7^ Department of Pediatrics UAMS Little Rock AR USA; ^8^ Arkansas Children’s Research Institute Little Rock AR USA

**Keywords:** 5/6 nephrectomy, chronic kidney disease, metaproteomics, microbiome, microbiota, resistant starch

## Abstract

**Background:**

Resistant Starch (RS) improves CKD outcomes. In this report, we study how RS modulates host‐microbiome interactions in CKD by measuring changes in the abundance of proteins and bacteria in the gut. In addition, we demonstrate RS‐mediated reduction in CKD‐induced kidney damage.

**Methods:**

Eight mice underwent 5/6 nephrectomy to induce CKD and eight served as healthy controls. CKD and Healthy (*H*) groups were further split into those receiving RS (*CKDRS*, *n* = 4; *HRS*, *n* = 4) and those on normal diet (*CKD*, *n* = 4, *H*, *n* = 4). Kidney injury was evaluated by measuring BUN/creatinine and by histopathological evaluation. Cecal contents were analyzed using mass spectrometry‐based metaproteomics and de novo sequencing using PEAKS. All the data were analyzed using R/Bioconductor packages.

**Results:**

The 5/6 nephrectomy compromised kidney function as seen by an increase in BUN/creatinine compared to healthy groups. Histopathology of kidney sections showed reduced tubulointerstitial injury in the CKDRS versus CKD group; while no significant difference in BUN/creatinine was observed between the two CKD groups. Identified proteins point toward a higher population of butyrate‐producing bacteria, reduced abundance of mucin‐degrading bacteria in the RS fed groups, and to the downregulation of indole metabolism in CKD groups.

**Conclusion:**

RS slows the progression of chronic kidney disease. Resistant starch supplementation leads to active bacterial proliferation and the reduction of harmful bacterial metabolites.

## Graphical abstract

During CKD, the gut microbiome was shown to produce nephrotoxins and compromise the epithelial barrier, contributing to the progression of the disease. Here, we investigate resistant starch‐induced gut microbiome changes in a 5/6 nephrectomy mouse model. Our results show a decrease in the development of kidney fibrosis upon RS supplementation.

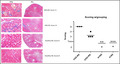

## 1 INTRODUCTION

Chronic kidney disease is an important problem affecting approximately 14% of the US population and leading to significant comorbidities like cardiovascular disease. Recent evidence shows that Chronic Kidney Disease (CKD) dramatically alters the gut microbiome, and this alteration further drives disease progression (Wing et al., 2016). Several factors are associated with the state of dysbiosis in CKD, including reduced dietary fiber consumption (Raj Krishnamurthy et al., 2012) and antibiotic treatment (Jernberg et al., 2007). The increase of toxic products in the blood that would be normally excreted in the urine leads to further progression of CKD and inflammation. Uremic toxins increase intestinal permeability, allowing greater absorption and contribution to dysbiosis (Vaziri, (2012)). Changes in the microbiome composition in CKD include the proliferation of urease‐containing species and depletion of microbes, fermenting carbohydrates. The fermentation results in short‐chain fatty acids (SCFAs)—butyrate, acetate, and propionate. Butyrate and other short chain amino acids are associated with the health of colonic epithelial and are an important source of energy for colonocytes. Their depletion is thought to lead to the disruption of the gut epithelial barrier. As a result, inflammatory mediators such as endotoxins leak into the bloodstream, contributing to the progression and complication of CKD. In addition, byproducts of bacterial metabolism, altered in CKD, such as indoxyl sulphate, p‐cresyl sulfate, trimethylamine N‐oxide (Tang et al., 2015; Vanholder & Glorieux, 2015; Vanholder et al., 2014) lead to renal injury and systemic inflammation.

Recently, gut microbiome modulation in CKD patients by oral supplementation with pre/pro/synbiotics has become a new avenue of research (Bliss et al., 1996; Rossi et al., 20162016; Viramontes‐Hörner et al., 2015). Generally, the conclusions from these studies were encouraging, namely that the use of pro/pre/synbiotics was decreasing uremic toxins and improving gastrointestinal symptoms (see (Armani et al., 2017) for review). However, Armani et al. (2017) suggests to interpret the results of the studies with caution because of their inherent limitations and notes that more studies are needed to evaluate the effect of pre/pro/synbiotics on gut microbiome modulation in CKD. In previous work from our group and others, it was shown that dietary supplementation by pre‐biotic fiber, resistant starch (RS) improves CKD outcomes in rats by increasing bacterial biomass and shifting composition toward butyrate‐producing bacteria (Kieffer et al., 2016; Zybailov et al., 2019). Furthermore, recent evidence from multiple sources suggests that supplementation with dietary fiber results in the restoration of the epithelial barrier integrity.

Despite the ameliorative effects of RS and other microbiome‐targeted therapies, trials in humans were ambiguous. In addition, most of the previous studies compared CKD animals, with and without supplementation, omitting healthy controls. In this work we used the 5/6 nephrectomy mice, using both healthy and CKD groups, to delineate effects of RS on kidney damage and function.

## 2 METHODS

### 2.1 Animal models

All experiments involving mice were conducted under an approved protocol overseen by the University of Arkansas for Medical Sciences Institutional Animal Care and Use Committee; Animal Use Approval File #3753. Sixteen male mice, 9 to 10 week old (weight 18‐20g) with a C57BL6 background were obtained from Jackson Laboratories (Bar Harbor, ME). After an initial week of acclimatization, eight mice underwent a two‐step surgical process (cortical electrocoagulation of 80% of the right kidney followed one week later by a left kidney total nephrectomy) to establish a reduced renal mass and induce CKD. As a CKD model we used the two‐step surgical process (cortical electrocoagulation of 80% of the right kidney followed two weeks later by a left kidney total nephrectomy), originally described by Gagnon et al. (Gagnon & Ansari, 1990) and validated by Shukla et al. (Shukla et al., 2015). All surgeries were performed under anesthesia and all efforts were made to minimize the pain and potential distress: meloxicam (an NSAID) at 1–2 mg/kg subcutaneously once a day pre‐operationally and for the next 2 days after surgery. Another eight mice served as healthy control.

Mice from two groups (CKD‐induced and healthy controls) were divided into four groups. Each group received semi‐purified pelleted diets either the regular diet (Harlan Laboratories) or a high‐fiber diet containing 59% high amylose maize resistant starch (HI‐MAIZE 260) for four weeks (*n* = 4 mice/diet), starting from the second postoperative day. As a result, there were four groups: CKD‐induced mice fed with regular diet, CKD‐induced mice fed with Resistant Starch diet, healthy mice fed with regular diet, healthy mice fed with Resistant Starch diet. All animals received semipurified pelleted isocaloric (3.4 kcal/g) diets with an energy content of 4.5%/66.9%/18.6% protein/carbohydrate/fat, respectively. Mice were housed three per cage and mice in the same cage were on the same diet. All animals were provided ad libitum access to food and water. The animals were then anesthetized (Ketamine/Xylazine IP) and euthanized via an overdose of carbon dioxide ((flow rate set to 10%–30% volume displacement per minute) followed by cervical dislocation.

After sacrificing, cecal contents were removed, frozen on dry ice, and stored at −70°C until processed.

Kidneys were excised and immersed in 10% Formalin overnight and then washed with PBS. First, all samples were ranked according to the time of sacrifice and experimental groups, bz‐1 to bz‐14. Next, a random sequence of 1–14 was generated and new indices ok‐1 through ok‐14 were reassigned to the samples. Only the first author of the paper—Oleg Karaduta had the index mapping key. Trichrome staining was performed according to the standard protocol [Carson, FL. Histotechnology: A Self‐Instructional Text, 2nd Edition]. Samples with new (randomly generated) indices were given to the third author of this paper—Zeljko Dvanajscak, a pathologist at ARKANA labs, who performed the scoring. Scarring usually begins in the subscapular region, but not always. It appears blue on trichrome. It extends vertically down in "columns" which are funnel shaped. When it atrophies enough, the distal medullary tubules start to dilate. All samples were arranged by Dr. Dvanajscak, from “most healthy” to “most damaged,” calibrated and given a score of either “zero,” “trace,” “one,” “two,” “three,” “three plus” according to renal pathology protocols. After the scoring was completed, the samples were re‐identified by the first author of this paper—Oleg Karaduta.

Kidney histology images were blindly scored by a board‐certified pathologist on a scale from 0 to 3+. Tubulointerstitial scarring was scored in a semiquantitative fashion, 0–3+, as for human renal biopsies. The scores were calibrated to severity based on a comparison among all the cases. The score included a combination of interstitial fibrosis and tubular atrophy (as for human renal biopsies), extending from the capsular surface through the cortex and into the medulla. Images were taken at a magnitude of 40χ and 400χ utilizing an Olympus BX53 microscope.

### 2.2 Soluble protein extraction

Briefly, 500 µl of PBS with protease inhibitor cocktail was added to the 100 mg of the frozen cecal content and vortexed vigorously for 5 min followed by sonication for 5 min. The supernatant (soluble proteins) was collected and protein concentration was determined using a BCA assay. The reproducibility of the extraction was evaluated by total de novo peptides identified and matched using online metaproteomics tool Unipept (Mesuere et al., 2015). Bacterial‐to‐host proteins ratio was used as an extraction/sample quality criteria.

### 2.3 Peptide identification and protein inference using database search

20 µg of protein for each sample was resolved on 4%–20% Tris‐Gly gel. The gels lanes were then cut in 24 pieces, in‐gel digested, and analyzed on a Thermo Scientific Orbitrap‐Tribrid‐Fusion instrument using the standard protocol at the UAMS Proteomics Core. Each SDS‐PAGE gel band was subjected to in‐gel trypsin digestion as follows. Gel slices were destained in 50% methanol (Fisher), 100 mM ammonium bicarbonate (Sigma‐Aldrich), followed by the reduction in 10 mM Tris[2‐carboxyethyl]phosphine (Pierce) and alkylation in 50 mM iodoacetamide (Sigma‐Aldrich). Gel slices were then dehydrated in acetonitrile (Fisher), followed by the addition of 100 ng porcine sequencing grade modified trypsin (Promega) in 100 mM ammonium bicarbonate (Sigma‐Aldrich) and incubation at 37°C for 12–16 hr. Peptide products were then acidified in 0.1% formic acid (Pierce). Tryptic peptides were separated by reverse‐phase XSelect CSH C18 2.5 um resin (Waters) on an in‐line 150 x 0.075 mm column using a nanoAcquity UPLC system (Waters). Peptides were eluted using a 30 min gradient from 97:3 to 67:33 buffer A:B ratio. [Buffer A = 0.1% formic acid, 0.5% acetonitrile; buffer B = 0.1% formic acid, 99.9% acetonitrile.]. Eluted peptides were ionized by electrospray (2.25 kV) followed by MS/MS analysis using higher‐energy collisional dissociation (HCD) on an Orbitrap Fusion Tribrid mass spectrometer (Thermo) in top‐speed data‐dependent mode. MS data were acquired using the FTMS analyzer in profile mode at a resolution of 240,000 over a range of 375 to 1,500 m/z. Following HCD activation, MS/MS data were acquired using the ion trap analyzer in centroid mode and normal mass range with precursor mass‐dependent normalized collision energy between 28.0 and 31.0.

For peptide identification and protein inference, a multi‐step database search strategy was used in PEAKS Studio to arrive at the final list of identified proteins. Files that were acquired on the Orbitrap Fusion Tribrid mass spectrometer in “.raw” format, were submitted to the de novo sequencing using PEAKS Studio v. 8 (Bioinformatics Solutions, Waterloo, ON, Canada). The following parameters were used for the data refinement: Merge Scans – left unchecked; Correct Precursor – mass only; Filter Scans – unchecked. The following parameters were used for the de novo sequencing: Parent Mass Error Tolerance – 5 ppm; Fragment Mass Error Tolerance – 0.5 Da; Enzyme – Trypsin; Fixed Modifications – Carbamidomethylation (C); Variable Modifications – Oxidation (M), Deamidation (NQ); Max Variable PTM Per Peptide – 3; Report # Peptides – 5.

### 2.4 Preliminary taxonomy analysis

De novo peptides identified by PEAKS were filtered using the average local confidence score (ALC%), and the peptides with ALC% above 80 were submitted to the online metaproteomics tool Unipept (Mesuere et al., 2015). In the Unipept/metaproteomics analysis tab the following parameters were set: Equate I and L ‐ checked, Filter duplicate peptides – unchecked, Advanced missed cleavage handling – checked. The taxonomy information was visualized using a tree diagram provided by Unipept.

For protein quantification, Scaffold v. 4 with a quantitation module (Proteome Software) was used. Data and the custom FASTA database were exported from PEAKS into Scaffold as mzIdentML and mascot generic format files. To analyze proteins: the total spectral counts for each protein were converted to NSAF values (Zybailov et al., 2006) using Scaffold, NSAF values were summed for identical proteins in different species, log2 transformed, and missing values imputed using Perseus (version 1.5.6.0, Max Planck Institute).

### 2.5 Differential abundance of proteins and bacteria between CKD, CKDRS, HRS, and H phenotypes

Spectral counts (number of tandem MS spectra that match to a given protein sequence via the database search) were used to infer differential abundant (DA) proteins and taxonomic units. At the taxonomic unit level, the spectral counts of proteins were grouped using taxonomic information in the sequence database and then were summed to obtain total spectral counts for each species in each sample. If species were not identifiable, higher taxonomic levels were used. The counts were filtered so that species with less than five counts in all samples but one was removed. Then counts were normalized to the trimmed mean of M values, a method frequently employed in RNA‐Seq analysis (Robinson & Oshlack, 2010).

Similar to metagenomics, there is no golden standard approach to identify differentially abundant proteins in metaproteomics. Recently it was shown that the NB distribution fits only about 90% of RNA‐seq expression profiles and is unable to fully capture the expression dynamic of RNA‐seq data (Esnaola et al., 2013). Instead of using “one‐fits‐all” NB distribution for fitting expression profiles, the authors (Esnaola et al., 2013) suggested to employ a more flexible Poisson‐Tweedy (PT) family of distributions. Given the similarity of metagenomics, metaproteomics, and RNA‐seq data distributional properties we decided to fit metaproteomics spectral counts to the general PT family (Esnaola et al., 2013). A two‐sample PT test is implemented in the Bioconductor package tweeDEseq (Esnaola et al., 2013). The raw counts were imported into R and analyzed using the tweeDE package. We implemented all six possible pairwise comparisons between four phenotypes and applied Benjamini‐Hochberg correction for multiple testing to define differentially abundant proteins and bacterial species (FDR < 0.05).

Heatmaps and hierarchical clustering of spectral counts were performed using the Bioconductor package pheatmap (https://cran.r‐project.org/web/packages/pheatmap/index.html). Proteins quantified across the different experimental groups (2,819 in total) are presented in Table S1a. Bacteria quantified across the different experimental groups (676 in total) are presented in Table S1b.

### 2.6 Network inference

To better understand the differences between CKD, CKDRS, HRS, and H phenotypes, we combined all bacteria that were differentially abundant in six comparisons to infer bacterial co‐abundance (BCoA) network. To infer the BCoA network, we used the BC3NET algorithm (Matos Simoes & Emmert‐Streib, 2012). This network inference method is a bagging version of C3NET (Altay & Emmert‐Streib, 2010) used to construct a mutual information‐based network. The C3NET algorithm has three main steps. First, it estimates mutual information between all pairs of entities (proteins or bacteria). Second, for each entity, the maximal mutual information pair is selected. Third, correction for multiple testing is applied. To visualize the most important connections within the inferred network, the network was reduced to a minimum spanning tree 2 (MST2) (see (Rahmatallah et al., 2017) for more details on MST2 trees). One of the most important network properties is its degree distribution, because it reveals highly connected nodes that define influential networks players. We defined highly connected nodes as those with a degree more than 95th percentile of the degree distribution. The inferred BCoA network had 149 nodes (bacterial species) and 405 connections, with giant component consisting of 120 nodes and 405 connections (i.e., 29 bacteria out of 149 were not connected). Average node's degree was 6.75 connections and average betweenness (the number of shortest paths going through the node) was 87.3. After reducing the network to an MST2 tree the average degree became 3.8 and average betweenness was the same. The 95th percentile of the degree distribution was 10.10 connections and as hub nodes, for further consideration, we selected only nodes with more than 10 connections. There were six bacteria with degrees more than 10: *Prevotella* sp.* CAG:873* (Mesuere et al., 2015), *Firmicutes bacterium ASF500* (50), *Robinsoniella* sp.* RHS* (23), uncultured *Roseburia* sp. (32), *Firmicutes bacterium CAG:65_45_313* (15), and *Oscillibacter* sp.* 1‐3* (12).

### 2.7 Data availability statement

Data are available via ProteomeXchange with identifier PXD019623.Submission details: Project Name: Investigation of Resistant Starch effect on gut microbiome in 5/6 nephrectomy mouse model of Chronic Kidney Disease. Project accession: PXD019623 Project https://doi.org/10.6019/PXD019623. Reviewer account details:

Username: reviewer98676@ebi.ac.uk.

Password: WSMSxJBO.

## 3 RESULTS

### 3.1 CKD model

Two‐step surgical process (cortical electrocoagulation of 80% of the right kidney followed two weeks later by a left kidney total nephrectomy) induces CKD. Both BUN and serum creatinine increased in the animals that underwent kidney mass reduction surgery (*p* < .05, Figure S1). However, there was no significant difference in BUN/Creatinine observed in CKD mice supplemented with RS compared to the CKD mice on a normal diet (Figure S1).

### 3.2 Histopathological analysis

**Figure 1:**
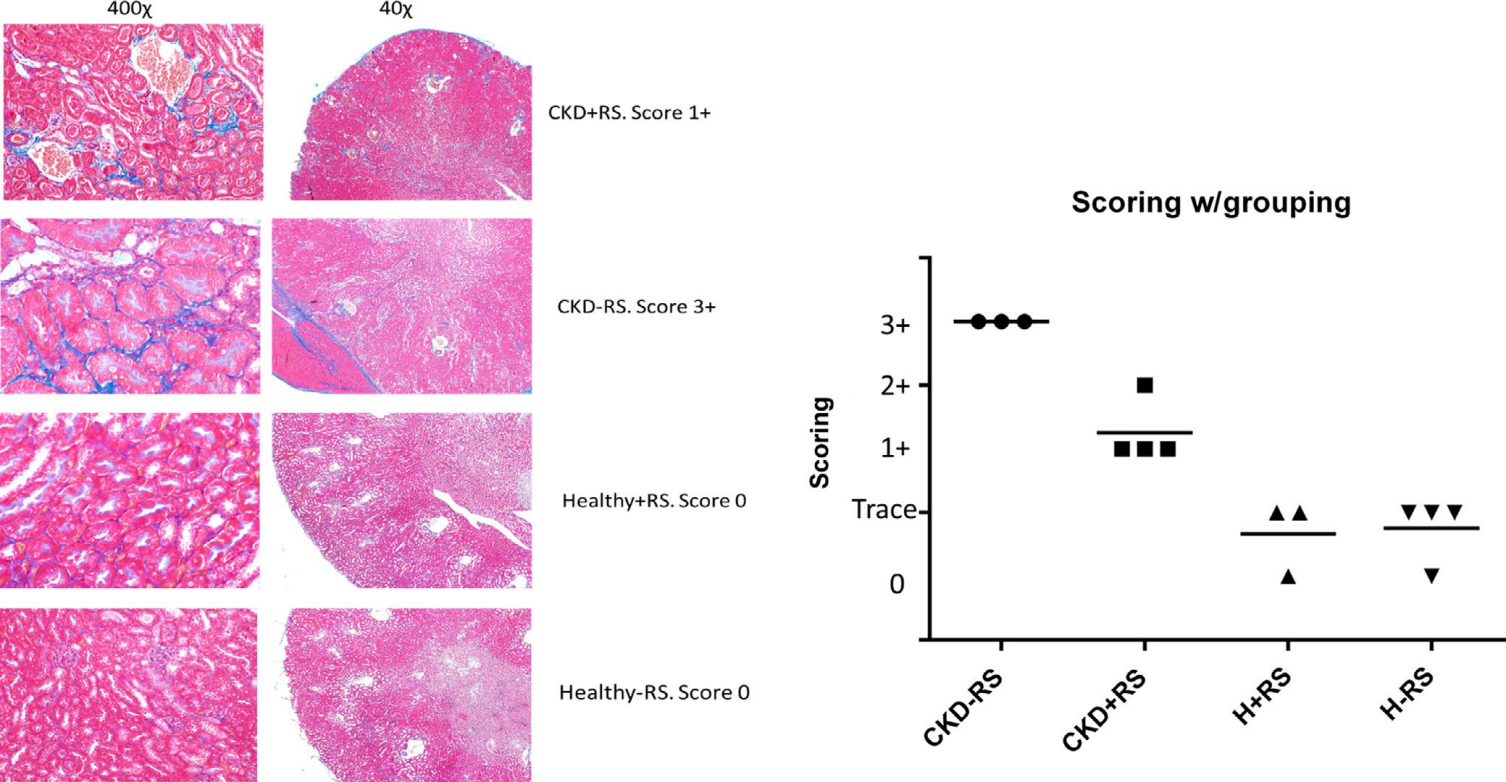
Histopathological analysis demonstrates reduced kidney damage in CKD mice supplemented with resistant starch. *Left* – representative photomicrographs of trichrome‐stained kidney sections. The kidney tissues in mice with surgically induced CKD showed severe tubulointerstitial injury marked by tubular atrophy and dilation, severe interstitial inflammatory cell infiltration, and interstitial fibrosis. The severity of tubulointerstitial injury in CKD rats fed HAM‐RS2 diet was significantly less than that found in the CKD rats fed low‐fiber control diet. *Right* – the results of histopathological scoring (also in Table 1). Higher scores indicate more severe kidney damage. The resistant starch‐fed CKD mice show scores in‐between disease and healthy groups

Representative photomicrographs of trichrome stained kidney sections are shown in Figure 1. The kidney tissues in mice with surgically induced CKD showed severe tubulointerstitial injury marked by tubular atrophy and dilation, severe interstitial inflammatory cell infiltration, and interstitial fibrosis. Histopathologic scoring of the severity of tubulointerstitial injury in CKD mice fed RS was significantly less than that found in the CKD mice fed low‐fiber control diet (Table 1).

**Table 1:** Scoring of the tubulointerstitial injury of the kidney in four experimental groups of mice

Mouse 1	CKD + RS	1+	CKD‐RS	3+	Healthy + RS	Trace+	Healthy‐RS	Trace+
Mouse 2	CKD + RS	2+	CKD‐RS	3+	Healthy + RS	0	Healthy‐RS	Trace+
Mouse 3	CKD + RS	1+	CKD‐RS	3+	Healthy + RS	Trace+	Healthy‐RS	Trace+
Mouse 4	CKD + RS	1+	CKD‐RS	*	Healthy + RS	*	Healthy‐RS	0

Note

CKDRS and * were not determined due to an insufficient quantity of material. See also Figure 1 for representative photomicrographs.

### 3.3 Differentially abundant proteins and bacteria

**Figure 2:**
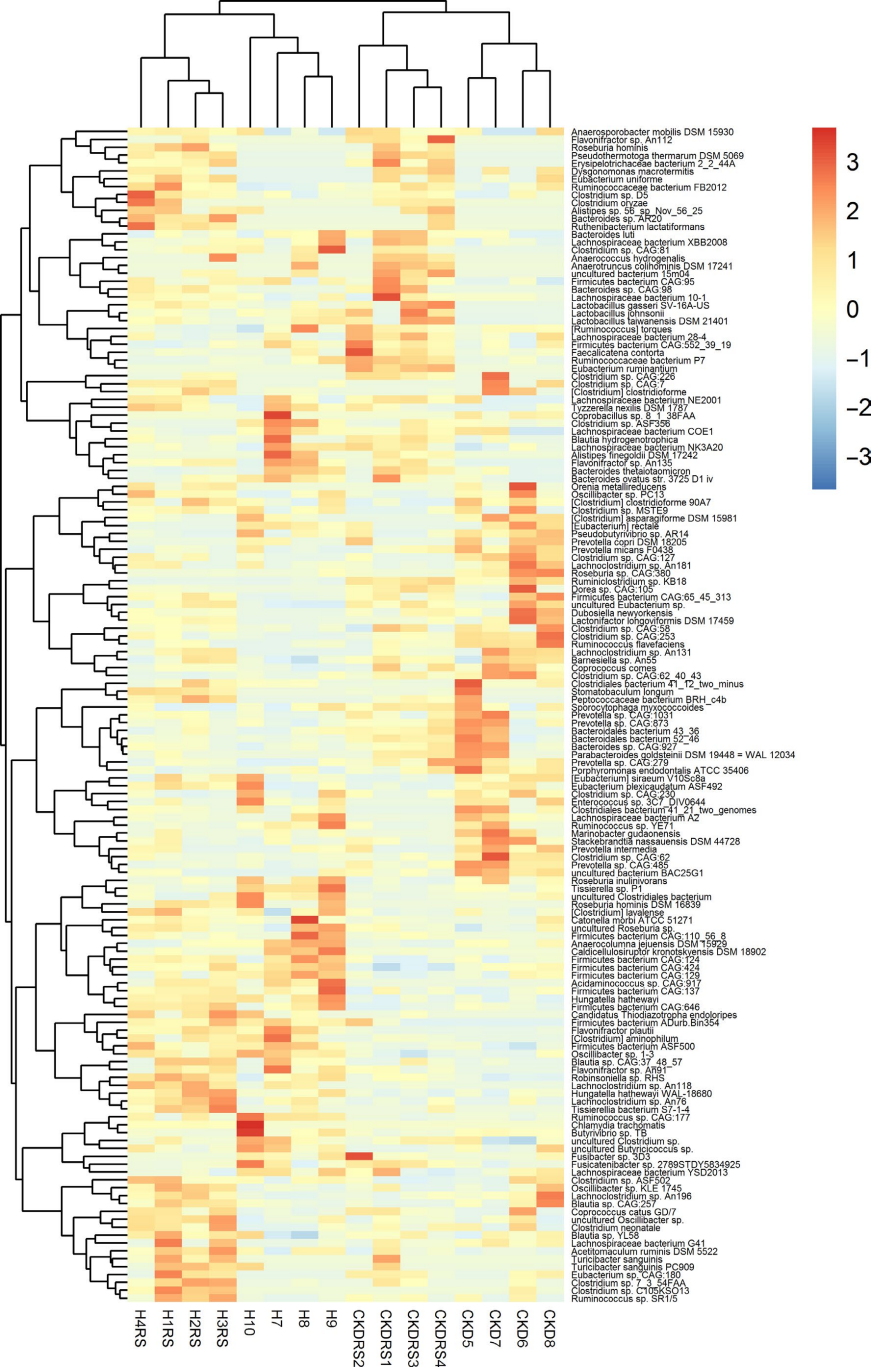
Bacterial abundance clearly delineates CKD from healthy phenotype, and also resistant starch from regular diet. Heatmap constructed from 149 differentially abundant bacteria (in at least one of the pairwise comparisons) is shown. Four phenotypes are indicated on the bottom labels—CKD, Healthy, with our without RS. Unsupervised clustering of bacterial abundance data results in the CKD and healthy mice grouped together, each further split into +RS and –RS groups

Figure 2 presents a heatmap, which was built using 149 unique differentially abundant (DA) bacteria from six pairwise comparisons between phenotypes (see Table S1b, for the full list of bacteria identified and compared across the experimental groups). When clustered according to the bacteria present the two dietary groups of CKD clustered together as did the two dietary groups that did not have CKD. In contrast, for 490 proteins, DA in six pairwise comparisons, clustering was different. Protein abundance clustered according to the dietary group instead of by disease groups. CKDRS and HRS formed one cluster and CKD and H formed another one (Figure S2). The most different phenotypes were HRS and CKD (38 GO categories, overrepresented in HRS as compared to CKD). That is, at the protein level with the addition of RS, CKD, and H, as well as HRS and CKDRS, were less different within groups, as compared to between groups, presumably because of the metabolic processes, specifically activated by RS in CKDRS and HRS phenotypes. Moreover, healthy gut microbiome was still more similar to healthy gut microbiome with the addition of RS, and CKD gut microbiome was still more similar to CKD gut microbiome even with the addition of RS.

Most of the mouse proteins that were DA between CKD and CKDRS were related to the adaptive immune response. Some of the mouse proteins, DA between CKD and CKDRS were also found in our previous study, for example, mucin (playing an important role in maintaining the integrity of the intestinal mucosal barrier), angiotensin‐converting enzyme (CKD biomarker), annexins A2 and A4 (cellular membrane repair) (Zybailov et al., 2019).

Several bacteria, DA between RS+ and RS‐ phenotypes were butyrate producers, similar to our previous study. For example, *Anaerococcus hydrogenalis* was more abundant in CKDRS as compared to CKD, *Roseburia hominis* was more abundant in HRS as compared to CKD and H phenotypes and *Coprococcus catus* GD/7 was more abundant in CKDRS and HRS as compared to H phenotype (the metabolic data for these strains were taken from the Virtual Metabolic Human (VMH) database (https://www.vmh.life/), capturing information on human and gut microbial metabolism). Butyrate producer *Eubacterium ruminantium* was present only in CKDRS phenotype and butyrate producer *Ruminococcus torques* was more abundant in CKDRS than CKD, similar to our previous results. Interestingly, it could be that the addition of RS to H or CKD phenotypes sometimes results in the proliferation of different butyrate producers (e.g., *Roseburia hominis* in HRS and *Eubacterium ruminantium, Ruminococcus torques* in CKDRS).

### 3.4 Blast2GO analysis

To define the enrichment of functional patterns associated with the differentially abundant proteins found by metaproteomics, we performed Gene Ontology analysis using Blast2Go (Conesa et al., 2005). The lists of bacterial proteins differentially abundant between CKD, CKD‐RS, HRS, and H phenotypes were tested for GO terms enrichment (biological process (BP), molecular function (MF), and cellular compartment (CC)) using Fisher exact test. All GO terms were considered significantly enriched if Fisher exact test p‐value was less than 0.01.

#### 3.4.1 CKDRS versus CKD

BPs overrepresented in CKDRS samples were inositol catabolic and metabolic processes, while MF was inositol 2‐dehydrogenase activity. There were no significant CC terms. Inositol catabolism can lead to butyrate production via a series of metabolic reactions sometimes encoded in microbial genomic structural variants (Zeevi et al., 2019). There were no overrepresented and up‐regulated GO terms in CKD samples at this significance level.

#### 3.4.2 HRS versus H

There were several overrepresented BPs in the comparison HRS versus H phenotypes (Table S3), as well as MFs (Table S4). Most processes, up‐regulated in HRS as compared to H phenotypes were related to glycolysis, anaerobe energy production pathway. Metabolism of branched‐chain amino acids was also up‐regulated in the HRS phenotype. The upregulation of those pathways is expected, because RS stimulates the proliferation of new bacteria and as a consequence, more energy and amino acids are required. There were no GO terms overrepresented in the comparison H versus HRS.

#### 3.4.3 CKDRS versus H

In the comparison between CKDRS versus H phenotypes the overrepresented BPs in CKDRS phenotype were related to energy production again, like in the case of HRS versus H, but through aerobic respiration (Table S5). GO BP terms, overrepresented in the comparison of H versus CKDRS were related to bacterial locomotion, indicating that in H phenotype there are, probably, more bacteria with flagella, as compared to CKDRS phenotype (Table S6). Overrepresented CC terms were also related to bacterial locomotion (Tables S7).

#### 3.4.4 CKD versus H

At the given significance level there was only one BP (cellular processes), overrepresented in CKD versus H comparison, and no BPs overrepresented in the H versus CKD phenotype.

#### 3.4.5 HRS versus CKD and HRS versus CKDRS

There were 38 BPs overrepresented in HRS as compared to the CKD phenotype and only 20 BPs overrepresented in the HRS phenotype as compared to CKDRS (Tables [Link], [Link] and [Link], [Link]), with 16 BPs in common between HRS versus CKD and HRS versus CKDRS. The 16 common processes included several general GO terms (e.g., cellular amino acid metabolic and catabolic processes, alpha‐amino acid metabolic and catabolic processes, cellular biogenic amine metabolic process) that are parent terms to more specific GO BP categories: tryptophan (Trp) metabolic and catabolic processes, indolalkylamine metabolic and catabolic processes and indole‐containing compound metabolic and processes. These BPs are interconnected and center on indole and tryptophan metabolism.

Processes, over‐represented in the HRS phenotype, as compared to CKDRS, similar to the comparison between HRS and H phenotypes, were related to energy production and carbohydrate metabolism. Processes, overrepresented in HRS as compared to CKD were branched‐chain amino acid metabolism, ketone body metabolic processes, and nitrogen catabolic processes. Ketone body metabolic processes are related to butyrate production and are expected to be up‐regulated in HRS as compared to CKD, due to the presence of RS degrading butyrate‐producing bacteria. Nitrogen catabolic processes are probably downregulated in CKD because of the presence of uremic toxins, and amino acid biosynthesis is presumably upregulated due to actively proliferating gut microbiome given RS feeding. Overall, the comparisons between HRS versus CKD and HRS versus CKDRS phenotype gave the most upregulated GO BPs, emphasizing the difference between those phenotypes, and revealing the downregulation of indole production in CKD and CKDRS phenotypes.

### 3.5 Bacterial co‐abundance (BCoA) network

**Figure 3:**
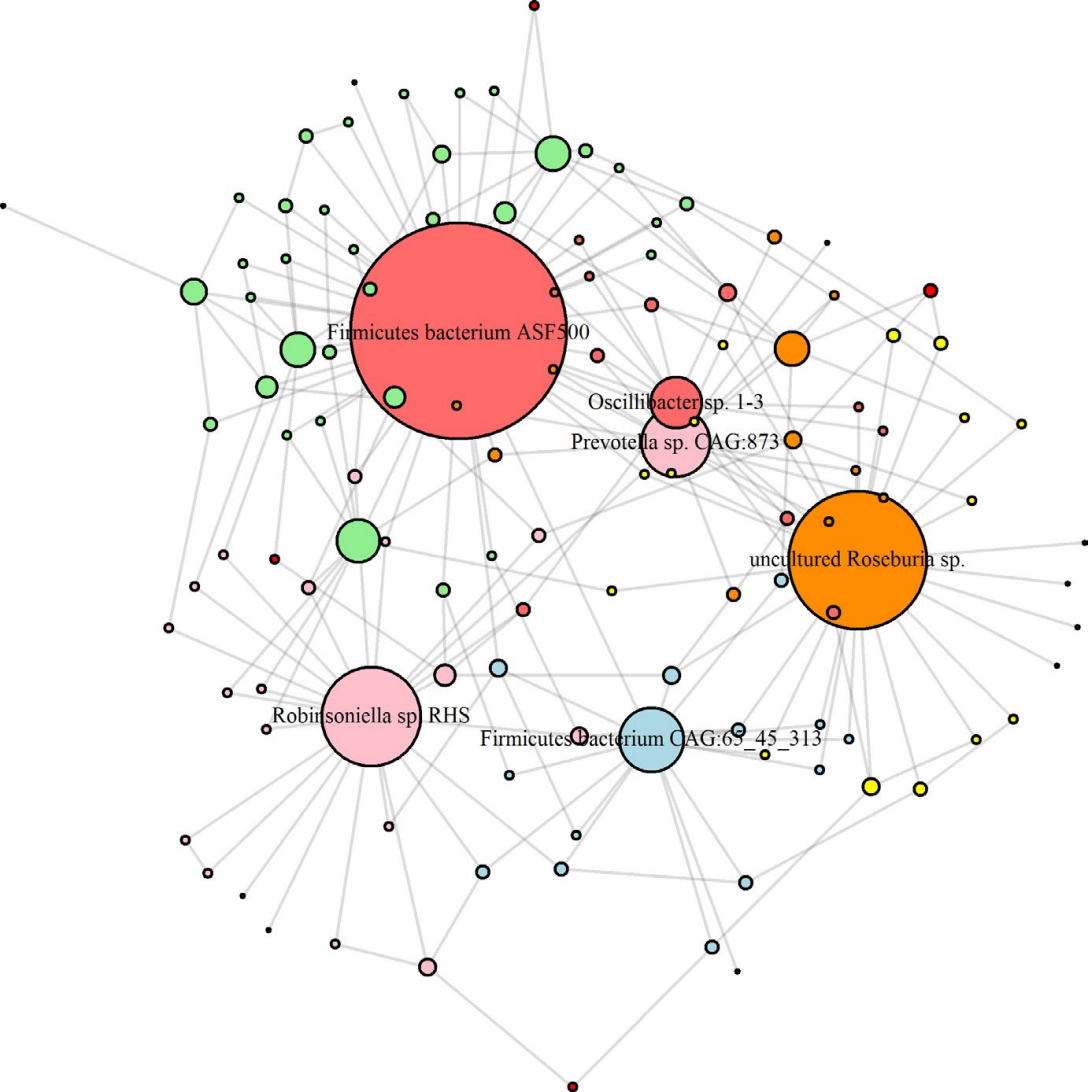
MST2 of bacterial co‐abundance (BCoA) network highlights bacteria functionally important in disease and RS metabolism. Co‐abundance network was inferred with bacteria, differentially abundant in six comparisons based on mutual information. Minimum spanning tree of the second degree was used to visualize functionally important hubs. As a result, six bacteria with degrees more than 10—*Prevotella* sp.* CAG:873*, *Firmicutes bacterium ASF500*, *Robinsoniella* sp.* RHS*, uncultured *Roseburia* sp., *Firmicutes bacterium CAG:65_45_313,* and *Oscillibacter* sp.* 1‐3* – are clearly visible in the network

Figure 3 presents the bacterial co‐abundance network inferred with bacteria, differentially abundant in six comparisons (see Table 2 and Methods section). The network consisted of 120 nodes and 405 connections (29 bacteria out of 149 were not connected) and the node size was selected to be proportional to node's degree. In this way six bacteria with degrees more than 10, namely *Prevotella* sp. CAG:873, *Firmicutes bacterium* ASF500, *Robinsoniella* sp. RHS, uncultured *Roseburia* sp., *Firmicutes bacterium* CAG:65_45_313, and Oscillibacter sp. 1–3 were clearly visible in the network (Figure 3). In what follows we call them “hub bacteria,” to underscore their importance in the network.

**Table 2:** Difference in bacterial abundances for six hub Bacteria for CKD, CKDRS, HRS, and H phenotypes (*t* test)

Hubs	*P*‐values, *t* test					
	CKD vs. CKDRS	CKD vs. H	CKD vs. HRS	CKDRS vs. HRS	CKDRS vs. H	HRS vs. H
*Firmicutes bacterium* ASF500	**.005**	**.025**	**.011**	**.044**	.107	.676
*Firmicutes bacterium* CAG:65_45_313	.955	.250	.705	.382	**.027**	**.019**
*Oscillibacter* sp. 1‐3	.419	**.022**	**.020**	**.038**	**.047**	.684
*Prevotella* sp. CAG:873.	.557	.182	.682	.199	**.029**	.147
*Robinsoniella* sp. RHS	**.012**	.089	**.005**	**.011**	.884	**.017**
uncultured *Roseburia* sp.	.087	.238	**.010**	.080	.754	.499

Note

The table shows p‐values for the pairwise comparisons of six hubs defined in Figure 2. *p*‐values below .05 are highlighted in *bold*.

**Figure 4:**
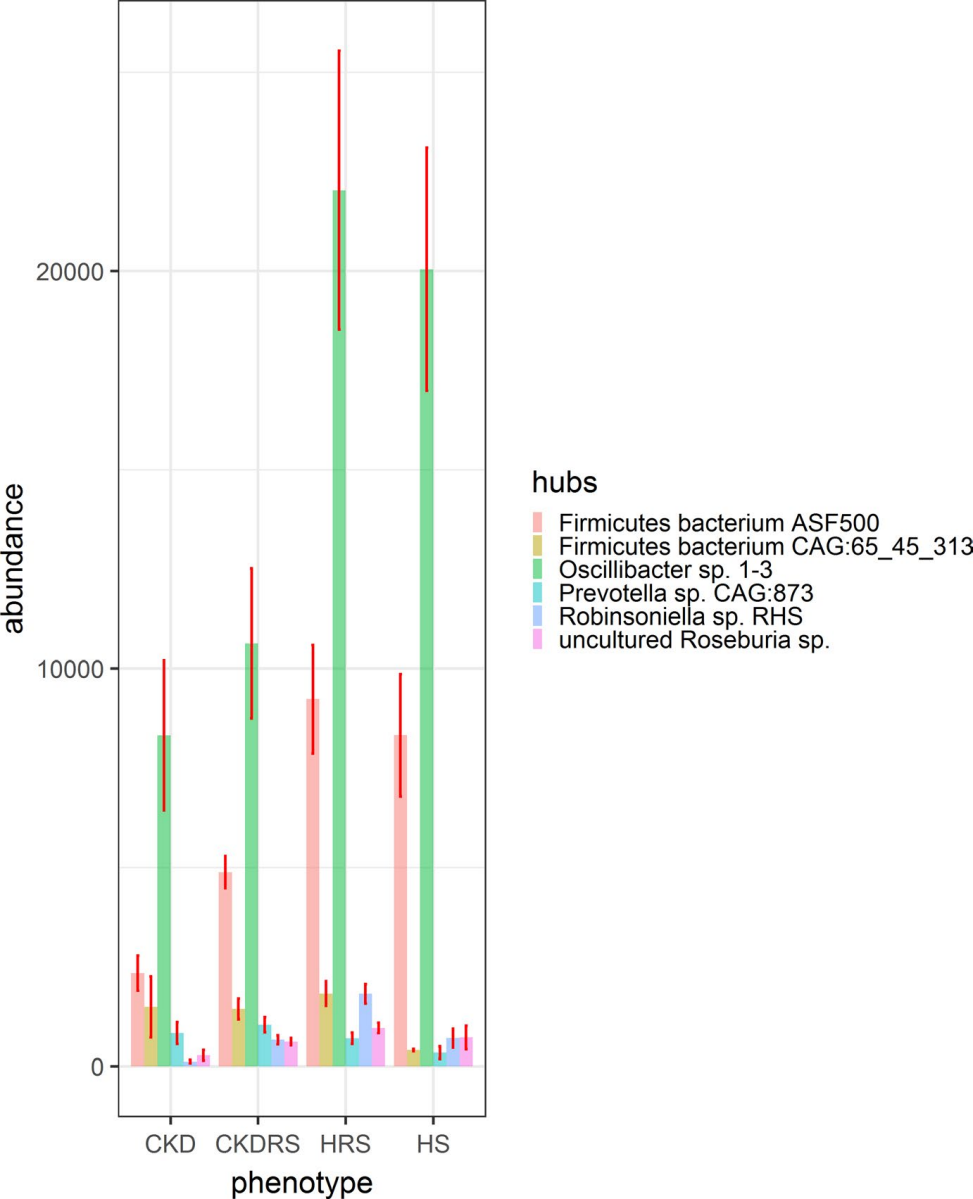
Abundances of hub bacteria in each phenotype. The bar plot shows abundances of the six bacteria highlighted in Figure 2. Abundances were inferred using summed and normalized spectral counts per each protein identified and mapped to a given bacteria. Hub bacteria are the least abundant in the CKD phenotype. At the same time, hub bacteria are more abundant in CKDRS phenotype as compared to CKD, as well as in the HRS phenotype, as compared to H

Figure 4 presents hub bacteria abundance in each phenotype. One can see that (1) overall, hub bacteria were the least abundant in the CKD phenotype and (2) hub bacteria were more abundant in CKDRS phenotype as compared to CKD, as well as in the HRS phenotype, as compared to H. That is, indeed, RS stimulates bacterial proliferation in all phenotypes, while chronic kidney disease condition decreases hub bacteria abundance in all phenotype (CKDRS and CKD have less hub bacteria than HRS and H).

Interestingly, the most abundant bacterium in the HRS phenotype was *Oscillibacter* sp. 1–3 (Figure 3), which contains tryptophanase, synthesizing indole from Trp. This result was consistent with Blast2GO analysis showing that Trp and indole metabolism were upregulated in the HRS phenotype as compared to CKDRS and CKD. *Oscillibacter* sp. 1–3 abundance was not significantly different between CKDRS versus CKD and HRS versus H phenotypes, but was significantly different between CKDRS, CKD versus HRS phenotypes (Table 2), in agreement with the result of Blast2GO analysis.

## 4 DISCUSSION

We and others have previously shown the effect of a resistant starch diet on CKD progression and the gut microbiome in CKD rats (Kieffer et al., 2016; Zybailov et al., 2019). There, we showed that the RS slows the progression of CKD and alters the gut microbiome. Here, we show that RS alters the gut microbiome in the 5/6 nephrectomy model of CKD in mice and that is associated with the reduction in kidney injury. Although BUN/Creatinine did not change significantly between the CKD‐RS and CKD mice, there was a clear difference in renal injury observed in the histopathological analysis between these two groups. The difference between CKD groups and Healthy control in creatinine and BUN was statistically significant, as expected. Increasing the number of animals per group in the future studies would likely show the difference in creatinine and BUN between the two CKD groups with and without starch. It is, however, clear the kidney function in the RS‐fed CKD group is still compromised. Yet, there is also evidence RS supplementation slows down the progression of the disease—it is clearly visible from Figure 1 where CKD + RS phenotype is exactly in between the CKD phenotype and healthy mice.

Consistent with our previous studies, we also observe a decrease in the Bacteroidetes‐to‐Firmicutes ratio in groups fed by the RS‐supplemented diet. Interestingly, both groups (CKD‐induced and healthy controls) reflect this tendency with around 1.5‐fold difference in the CKD‐induced group of mice and 8% in healthy controls. Alpha diversity indices were similar between healthy control and CKD mice on a regular diet (results not shown). In healthy control mice, RS induced a decrease of alpha diversity indices. In CKD mice, a significant increase in species richness was observed in CKD mice fed with RS‐supplement. At the phylum level, no difference was observed between healthy control mice and CKD mice on a regular diet at the end of week 4.

The inclusion of healthy controls allowed us to see metabolic processes and bacteria, activated by RS in CKD, CKDRS phenotypes, as compared to H, HRS controls. In particular, upregulation of indole metabolism and catabolism in the HRS phenotype, as compared to CKD and CKDRS phenotypes (not in the comparisons of H versus HRS) is a new finding that was not observed before. Trp metabolism in the gut consists of direct Trp transformation into indole and its derivatives by bacteria that express tryptophanase. Indole is synthesized from Trp by tryptophanase in a large number of Gram‐positive and Gram‐negative species. Indole is the most abundant microbial catabolite in healthy adults, following by indoleacetic acid (IAA) and indolpropionic acid (IPA) (Esnaola et al., 2013). Indole per se can be beneficial and is considered to be an intercellular signaling molecule with a wide spectrum of activities, including anticancer, anti‐inflammatory, antimicrobial, and antidiabetic (Florens et al., 2017). Also, indole enhances intestinal epithelial barrier functions by increasing the expression of genes involved in the maintenance of epithelial cell structure and function (Andrikopoulos et al., 2005; Feng et al., 2015). It was also suggested that the manipulation of indole concentrations in the gastrointestinal tract by pre‐ or probiotics that produce indole can limit the virulence of enteric pathogens (Rajilić–Stojanović et al., 2011). However, some of its derivatives, such as indoxyl sulfate (indole converted to indoxyl sulfate (IS) by the host), indole‐3‐acetate, and indole‐3‐lactate can be potentially harmful and are uremic retention solutes (URS) that accumulate in the blood and tissues of CKD patients (Ramezani & Raj, 2014). It was previously shown that RS‐fed rats had decreased level of IS in the urine and serum, and increased level of indole‐3‐acetate and indole‐3‐lactate in serum, despite unchanged or reduced levels in the cecum and urine, was also observed (Ramezani & Raj, 2014). Our result did show increased indole metabolism and catabolism in the HRS phenotype and their decrease in CKD and CKDRS phenotypes. In other words, in the HRS phenotype, Trp metabolism and catabolism result in more indole production and indole utilization than in CKD and CKDRS phenotypes. A simple mechanistic explanation of the observed phenomena would be that the HRS phenotype contains more Trp metabolizing indole‐producing bacteria as compared to CKD and even CKDRS phenotypes. We did observe the increase of at least one indole‐producing bacterial species. As it was already mentioned indole has beneficial and potentially harmful (e.g., IS) properties, however, its most beneficial effects for the CKD phenotype would be the upregulating of expression of tight junction proteins and modulation of expressions of pro‐ and anti‐inflammatory genes in intestinal epithelial cells. It was suggested that diverting tryptophan metabolism away from IndS toward IPA would be beneficial in renal disease (Zhang & Davies, 2016). Presumably, RS supplementation could be an option. However, how exactly in the HRS phenotype, the proper balance between beneficial and harmful properties of indole is achieved remains to be studied. In the present study, CKD mice exhibited impaired kidney function consistent with previous publications. Dietary RS was associated with amelioration of renal histopathological abnormalities.

Notably, all mice that underwent surgeries received NSAID on the first post‐operational day. Both NSAID as well as surgeries themselves can potentially effect gut microbiome. We believe it is unlikely that a single NSAID injection would lead to significant changes. Still, it would be interesting to compare the surgical model of CKD to a chemical model, where CKD is induced by adenine supplementation to address these potentially confounding effects on microbiome.

CKD has been shown to be associated with intestinal microbial imbalance. RS may be an excellent strategy to re‐establish gut symbiosis as gut microbiota is the primary target of dietary fiber. In our study, alpha diversity was similar in control and CKD mice, and dietary RS increased alpha diversity (much more prominently in the control mice). In general, the alpha diversity for bacterial richness is considered a major marker for gut health and often reflects gut stability and resilience. However, another study showed greater gut microbiota richness in colon cancer patients compared to healthy subjects (Feng et al., 2015).

Therefore, microbial function and bacterial composition are more important than diversity in regulating host physiology. In addition, RS significantly reduced the abundance of six out of nine CKD‐associated genera. The abundance of the genera *Clostridium* and *Staphylococcus*, which include several well‐known opportunistic pathogens, was about significantly higher in CKD mice compared to healthy control mice. Abundance of the genus *Dorea*, which has been reported to be significantly increased in IBD patients (Rajilić–Stojanović et al., 2011), was about 3‐fold higher in CKD mice. *Lactobacillus* has been reported to decrease and is considered beneficial in patients with CKD (Ramezani & Raj, 2014). Further taxonomic subdivision of the genus *Lactobacillus* showed that changes are within unclassified species, and do not include *Lactobacillus* species with known probiotic effects. *Erysipelotrichaceae* is known to be highly immunogenic and has been associated with IBD (Kaakoush, 2015). Its abundance is also higher in CKD mice compared to control mice. RS supplementation showed dose‐dependently decreased it in CKD mice.

At the species level, we found that *Firmicutes bacterium ASF500* was significantly more abundant in CKDRS as compared to the CKD phenotype. It belongs to the *altered Schaedler flora* (ASF), a bacterial community that supports normal growth and development of gnotobiotic mice (Wannemuehler et al., 2014). It was recently shown, that the adhesion of specific members of the gut microbiome to intestinal epithelial cells is essential for the induction of Th17 cells (Atarashi et al., 2015). In addition to segmented filamentous bacteria (SFB), one of the most potent inducers of Th17 cells, a mixture of 20 bacterial strains was also able to cause robust induction of Th17 cells, with *Firmicutes bacterium ASF500* being one of the mixture's member (Atarashi et al., 2015). Th17 cells are critical in protecting mucosal surfaces against microbial pathogens, in concert with other immune cells (Omenetti & Pizarro, 2015), as well as, in protecting the intestinal barrier (Stockinger & Omenetti, 2017). It is highly plausible that the deficiency of *Firmicutes bacterium ASF500* may contribute to the CKD dysbiosis phenotype; however, it is important to note that bacteria do not act in isolation. Concordantly, at the protein level, we found several members of immunoglobulin families (e.g., Ighv1‐39, Ighv1‐81, Ighv3‐3, Ighv1‐53, Igh) that were significantly more abundant in CKDRS than CKD.

*Robinsoniella* sp.* RHS* was significantly different between CKD versus CKDRS, CKD versus HRS, and HRS versus H phenotypes, being the most abundant in the HRS phenotype. Bacterium *Robinsoniella* sp.* RHS* was shown to be highly competitive in colonizing mouse gut (Seedorf et al., 2014) and was also recovered from swine and human feces (Cotta et al., 2009; Gomez et al., 2011), with the major end products of metabolism included acetate and succinate but not butyrate (Cotta et al., 2009).

The two bacteria that were the most significantly different (in four comparisons out of six) between phenotypes were *Oscillibacter* sp.* 1‐3* and *Firmicutes bacterium ASF500*. They were twice as abundant in the HRS phenotype as compared to the CKD phenotype and both of them have important properties in enhancing intestinal epithelial barrier functions and immunity.

In summary, our study is the first to demonstrate a slowdown in the development of renal injury in CKD mice upon RS supplementation. Also, we show that the benefits of RS reach beyond the enhancement of butyrate production: the comparison with healthy controls shows increased indole metabolism and increase in bacteria, related to gut health.

## CONFLICT OF INTEREST

None.

## AUTHOR CONTRIBUTIONS

OK and BZ designed the study; LY developed the animal protocol, OK carried out experiments; SM, LO, and AT carried out mass spectrometry experiments. GG and YR analyzed the data and made the figures; BZ and JA revised the manuscript; ZD performed staining and scoring of kidney samples.

## Supporting information



Fig S1‐S2

Table S1

Table S2

Table S3‐S9

Table S10

Table S11
