# Supplementary material for: Resistant starch slows the progression of CKD in the 5/6 nephrectomy mouse model
Source: Physiol Rep. 2020 Oct 10;8(19):e14610. doi: 10.14814/phy2.14610 (PMC7547583; doi:10.14814/phy2.14610)
Supplement: Supplementary file 1 — Fig S1‐S2 [file PHY2-8-e14610-s001.pdf]

Supplementary Materials

SupplementaryTableS1. Quantifiable proteins and quantifiable taxonomic units.

SupplementaryTableS2. Mouse proteins, differentially abundant in six comparisons.

SupplementaryTableS3-9. GO analyses.

SupplementaryTableS10. A six-way Venn diagram of the bacteria that are significantly differentially abundant between 6 phenotypes.

Supplementary Table S11. Bacteria, the most abundant in six pairwise comparisons.

Supplementary Figure 1. BUN and creatinine levels in mice after 5/6 nephrectomy.

Supplementary Figure 2. Heatmap of identified proteins clustered by abundance values.

	BUN						Creatinine					
replicate#	1	2	3	4	mean	stdev	1	2	3	4	mean	stdev
HRS	13.7	11.2	7.5	11.6	11	2.578113	0.41	0.51	0.37	0.68	0.4925	0.138173
H	17.3	15.2	17.1	6.7	14.075	5.006912	0.47	0.53	0.52	0.44	0.49	0.042426
CKD	16.487	57.844	35.977	42.218	38.1315	17.11148	9	5.328	9	6.387	7.42875	1.865123
CKDRS	72.434	64.797	58.452	13.273	52.239	26.59882	3.354	3.695	4.774	6.743	4.6415	1.526147

Figure S1

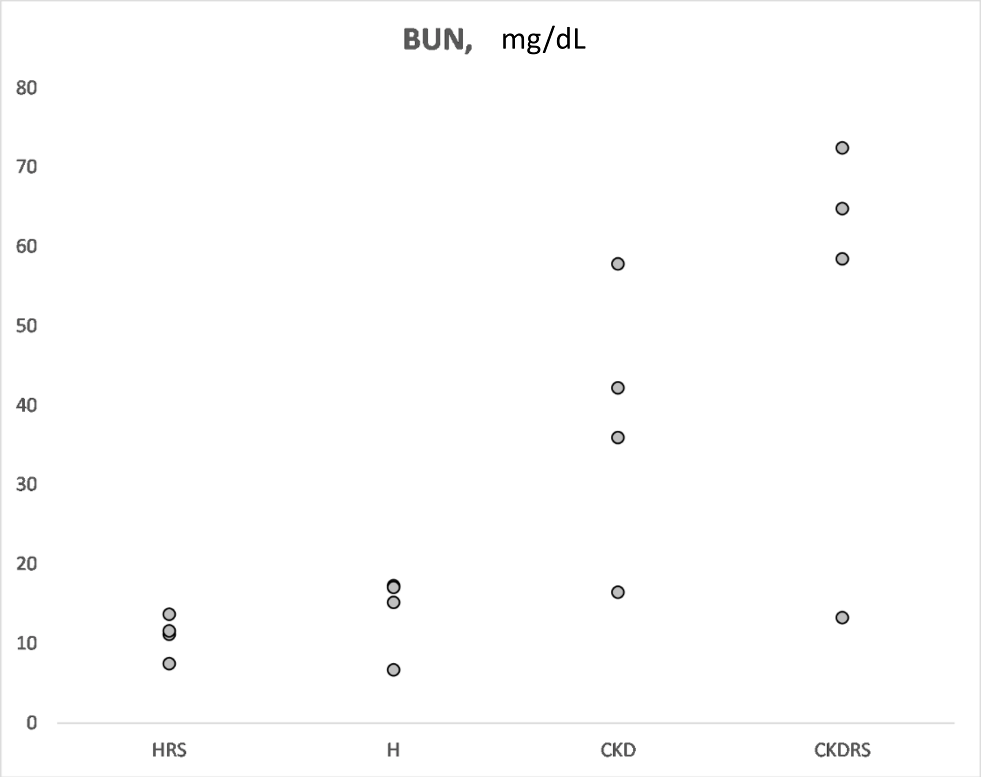

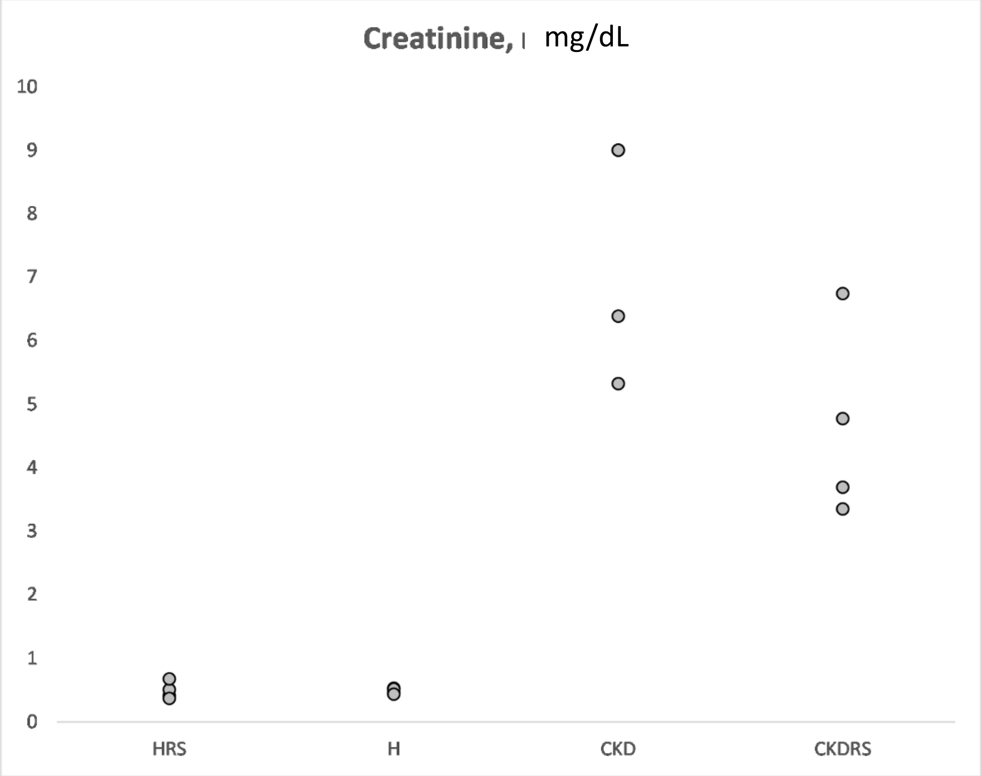

p-values (ttest)				
	HRS	H	CKD	CKDRS
HRS	1			
H	0.316715	1		
CKD	0.003693	0.035643	1	
CKDRS	0.021488	0.030351	0.40669	1

p-values (ttest)				
	HRS	H	CKD	CKDRS
HRS	1			
H	0.973526701	1		
CKD	4.55453E-07	7.94915E-05	1	
CKDRS	0.001640392	0.001604778	0.364497	1

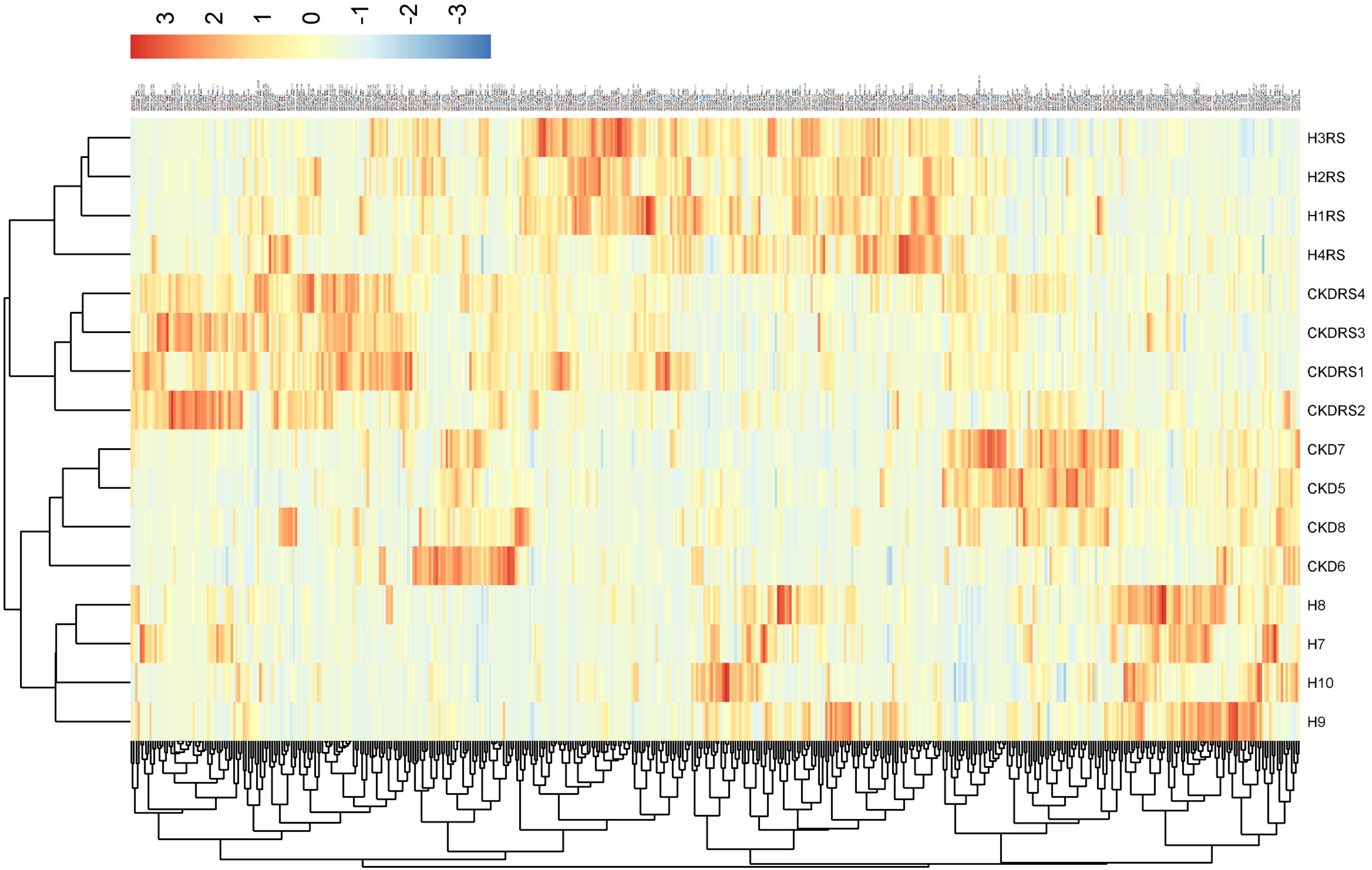
3
2
1
0
-1
-2
-3
H3RS
H2RS
H1RS
H4RS
CKDRS4
CKDRS3
CKDRS1
CKDRS2
CKD7
CKD5
CKD8
CKD6
H8
H7
H10
H9

Figure S2
